# Primary Uterine NUT Carcinoma: A Case Report and Literature Review

**DOI:** 10.3390/clinpract16010020

**Published:** 2026-01-21

**Authors:** Tetsuro Shiraishi, Iori Kisu, Naomi Kaneko, Takaaki Fukuda, Jun Watanabe, Ryoma Hayashi, Akihisa Ueno, Katsura Emoto, Kanako Nakamura, Yuya Nogami, Kosuke Tsuji, Kenta Masuda, Wataru Yamagami

**Affiliations:** 1 Department of Obstetrics and Gynecology, Keio University School of Medicine, Tokyo 160-0016, Japan; t.shiraishi@keio.jp (T.S.); 2 Department of Gynecology, National Cancer Center Hospital, Tokyo 104-0045, Japan; 3 Department of Pathology, Keio University School of Medicine, Tokyo 160-0016, Japan

**Keywords:** nuclear protein in testis (NUT) carcinoma, uterine corpus cancer, *NUTM1*-fusions, undifferentiated carcinoma, uterine sarcoma

## Abstract

**Background**: Nuclear protein in testis (NUT) carcinoma is a rare, aggressive, and poorly differentiated epithelial malignancy characterized by the rearrangement of *NUTM1* (NUT midline carcinoma family member 1) on 15q14. It primarily originates along the midline structures, including the head, neck, thorax, and mediastinum. Although NUT carcinoma of the pelvic gynecological organs is exceedingly rare, reported cases have been limited to primary or metastatic ovarian tumors. Here, we present the first documented case of primary uterine NUT carcinoma. **Case presentation**: A 53-year-old postmenopausal woman presented with abnormal uterine bleeding and a uterine mass. She underwent a total abdominal hysterectomy with bilateral salpingo-oophorectomy. The initial postoperative histopathological evaluation suggested undifferentiated endometrial sarcoma; however, subsequent immunohistochemical (IHC) analysis and fluorescence in situ hybridization revealed *NUTM1* rearrangement, confirming the diagnosis of NUT carcinoma. The patient experienced tumor recurrence six months postoperatively and succumbed to the disease nine months later. **Discussion**: The pathological diagnosis was challenging; the presence of abrupt squamous differentiation prompted further IHC analysis, leading to the definitive diagnosis. Primary uterine NUT carcinoma may be misdiagnosed as other undifferentiated uterine tumors due to its rarity and histological overlap. **Conclusions**: Given the diagnostic challenges, NUT IHC staining and molecular testing for *NUTM1* rearrangement should be considered in undifferentiated uterine tumors with ambiguous histopathological features.

## 1. Introduction

Nuclear protein in testis (NUT) carcinoma is a rare, highly aggressive, and poorly differentiated epithelial malignancy characterized by the rearrangement of *NUTM1* (NUT midline carcinoma family member 1) on 15q14 [1]. It was first described in 1991 as a thymic carcinoma in young adults with a t(15;19)(q15;p13) translocation [2]. Since its initial identification, NUT carcinoma has been increasingly recognized through numerous case reports. Although it primarily arises along the midline of the body, most commonly in the head and neck, thorax, and mediastinum, it has also been reported in extramidline sites, including the lung, salivary glands, pancreas, bladder, kidney, adrenal glands, ovary, and bone [1,3,4]. Although NUT carcinoma predominantly affects adolescents and young adults, it can occur at any age, ranging from infancy to the eighth decade of life [5]. The prognosis remains extremely poor, with a median overall survival of only 6.7 months in the absence of a standardized effective therapy [6].

Only a few cases of NUT carcinoma involving pelvic gynecological organs have been reported, all of which were either primary or metastatic ovarian NUT carcinomas [4,7,8,9,10]. To the best of our knowledge, this study presents the first reported case of primary uterine NUT carcinoma.

## 2. Case Report

**Figure 1:**
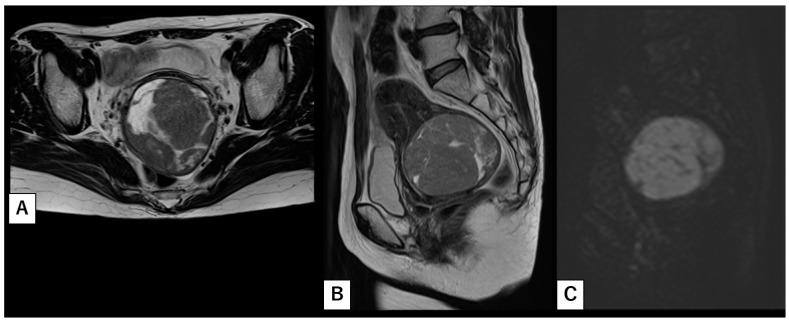
Pelvic MRI findings (**A**,**B**). Pelvic MRI (T2 weighted) reveals a 7.4-cm mass situated within the posterior wall of the lower uterine body. (**C**) Diffusion-weighted imaging (DWI) shows a high signal within the cystic component, suggestive of degenerative, necrotic, or malignant tissue.

A 53-year-old postmenopausal female (para 2) presented with abnormal uterine bleeding and was referred to our institution with a suspected uterine fibroid or sarcoma. Her past medical history was unremarkable except for a previous surgical abortion. Pelvic magnetic resonance imaging (MRI) revealed a 7.4-cm mass located within the posterior wall of the lower uterine body (Figure 1A,B). Diffusion-weighted imaging (DWI) demonstrated a high signal within a cystic component, suggestive of degenerative, necrotic, or malignant tissue (Figure 1C). Given the well-defined borders of the mass and the absence of overt invasion or metastasis, our initial differential diagnosis included cellular leiomyoma and low-grade endometrial stromal sarcoma. Laboratory tests, including tumor markers (LDH 173 U/L, CEA 1.3 ng/mL, CA 19-9 13 U/mL, and CA-125 7 U/mL), were within normal limits, and both endocervical and endometrial smears were negative. Endometrial biopsy did not indicate malignancy.

**Figure 2:**
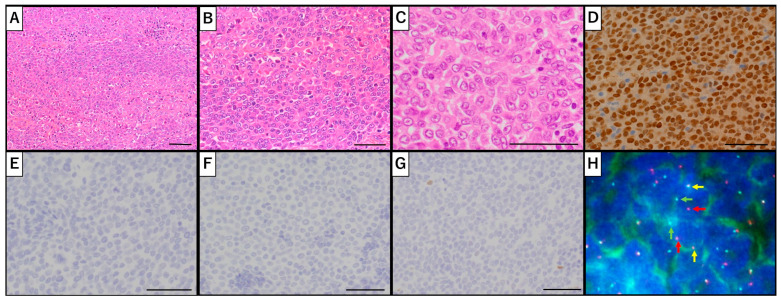
Molecular pathological findings (**A**) Uniformly spherical tumor cells proliferate in a solid pattern (×200, Bar = 50 µm). (**B**) Tumor cells demonstrate centrally located, enlarged nuclei and prominent nucleoli (×400, Bar = 50 µm). (**C**) Focal squamous differentiation is noted, representing less than 5% of the tumor (×400, Bar = 50 µm). (**D**) Both diffuse and strong NUT expression were observed (×400, Bar = 50 µm). The positive control was from testis tissue, whereas the negative control was non-cancerous tissue free of *NUTM1* mutations. (**E**) AE1/AE3 and (**F**) p40 are negative, whereas (**G**) p63 expression is rare (×400, Bar = 50 µm). (**H**) The *NUTM1*-break-apart signals on fluorescence in situ hybridization (FISH). NUT carcinoma revealed one green/red fusion signal (yellow arrows) and one green/red separate signal (green/red arrows), indicating *NUTM1*-rearrangement. We used the NUTM1 Break Apart FISH Probe (Empire Genomics, Buffalo, NY, USA) according to the manufacturer’s protocols, which was labeled in green on the 5′-side and red on the 3′-side of *NUTM1* (15q14).

Approximately 3 months after the initial referral, the patient underwent an abdominal total hysterectomy with bilateral salpingo-oophorectomy. Intraoperatively, the uterine mass was mobile and free of adhesions, extrauterine invasion, or metastasis. Histopathological evaluation revealed uniformly round tumor cells with moderate amounts of amphophilic cytoplasm and centrally located, enlarged nuclei with prominent nucleoli arranged in a loosely cohesive pattern (Figure 2A,B). Although the tumor invaded deeply into the myometrium, no evidence of extrauterine extension was observed. A broad IHC panel was performed to evaluate various uterine tumors; however, the findings remained inconclusive, with undifferentiated carcinoma, high-grade endometrial stromal sarcoma, and undifferentiated uterine sarcoma all being considered (Table 1). Subsequent review in collaboration with the pathology team revealed focal squamous differentiation (Figure 2C), prompting additional immunostaining for NUT, which demonstrated diffuse positivity (Figure 2D). Moreover, *NUTM1*-break-apart signals were confirmed by fluorescence in situ hybridization (FISH), which genetically proved *NUTM1*-rearrangement of the tumor (Figure 2H). Given the possibility that the NUT carcinoma might have originated from other sites (e.g., head and neck or thorax), a fluorodeoxyglucose positron emission tomography (FDG-PET) scan was performed; however, no abnormal FDG uptake suggestive of a primary or residual tumor was identified. Thus, a final diagnosis of primary uterine corpus NUT carcinoma was established. Ascites cytology was negative.

Considering the lack of effective postoperative treatments for NUT carcinoma, the patient was closely monitored with follow-up visits every 2 months. Six months postoperatively, small nodules were detected on the vaginal wall and pelvic floor; pathological analysis confirmed recurrent NUT carcinoma. FDG-PET further revealed disseminated nodules in the abdominal and retroperitoneal cavities, pleural metastases with associated effusions, and multiple mediastinal and cervical lymph node metastases. A computed tomography scan of the brain did not reveal any metastatic lesions. Comprehensive genomic profiling was proposed to identify potential therapeutic options; however, acknowledging the grim prognosis, the patient declined further invasive diagnostics and opted to focus solely on palliative care. Following a unilateral percutaneous nephrostomy performed for ureteral stricture secondary to pelvic seeding, she was transferred to a palliative care unit at another institution. The patient ultimately succumbed to her disease 15 months after the initial surgery.

## 3. Discussion

NUT carcinoma is well recognized in head and neck surgery; however, its occurrence in gynecology remains exceptionally rare. We conducted a literature review for NUT carcinoma cases in pelvic gynecological organs, including case reports, systematic reviews, meta-analyses, randomized controlled trials, and clinical trials. Various databases were consulted: all cases from Web of Science overlapped with those from PubMed, and no cases were identified from Cochrane Library (Supplementary Figure S1). Currently, only six cases of primary or metastatic ovarian NUT carcinoma have been documented (Table 2) [4,7,8,9,10,11]. To the best of our knowledge, this report represents the first case of primary uterine NUT carcinoma.

A preoperative suspicion of NUT carcinoma was Impossible. In our patient, a non-contrast MRI, performed owing to her history of asthma, suggested cellular leiomyoma or low-grade endometrial stromal sarcoma. Although gadolinium-enhanced imaging might have accentuated necrotic features within the mass, such findings could nonetheless have been interpreted as benign. In addition, normal blood tests and negative results from cytology and endometrial biopsy contributed little to clinical suspicion, leaving only intermittent uterine bleeding and a high signal on DWI as indicators of an underlying malignancy. In the absence of distant metastasis or lymphadenopathy, diagnostic surgery became the sole method to establish a definitive diagnosis.

Postoperative pathological evaluation further underscored the diagnostic challenges. NUT carcinoma typically exhibits nests and sheets of primitive round cells with prominent nucleoli, enlarged nuclei, frequent mitoses, and areas of necrosis, features that were present in our case [5]. Regarding IHC in typical NUT carcinoma, cytokeratins (AE1/AE3, CK7, CK20) are expressed in 75% of the cases, whereas p63 or p40 are observed in nearly 50%, consistent with squamous cell carcinoma. However, this indicates that cytokeratins and p40/p63 can be negative, as observed in our case. Moreover, in some cases, neuroendocrine markers (e.g., S100, synaptophysin, chromogranin A) are reported to be positive [12]. In our case, an extensive panel of IHC stains initially suggested a diagnosis of undifferentiated carcinoma, high-grade endometrial stromal sarcoma, or undifferentiated uterine sarcoma, yet none yielded a definitive diagnosis. It was only after obtaining a second pathological opinion from a specialist oncology center—where abrupt squamous differentiation, a hallmark of NUT carcinoma, was identified—that additional NUT staining was performed, revealing diffuse positivity. The NUT test is an important basis for diagnosis, showing 87% sensitivity, nearly 100% specificity, a negative predictive value of 99%, and a positive predictive value of 100% [12]. Given that squamous differentiation is observed in only about 30% of NUT carcinoma cases [13], routine NUT IHC staining is crucial when evaluating poorly differentiated malignant uterine neoplasms. Based on our experience, it is possible that some tumors previously classified as undifferentiated or dedifferentiated carcinoma, high-grade endometrial stromal sarcoma, or undifferentiated uterine sarcoma may indeed represent NUT carcinoma. Although no established treatment exists, the dismal prognosis associated with NUT carcinoma mandates prompt treatment decisions grounded in accurate diagnosis.

At the molecular level, NUT carcinoma is characterized by rearrangements of the *NUTM1* gene. Recent genomic profiling has revealed that most *NUTM1* fusions involve *BRD4* (bromodomain-containing protein 4) on 19p13.1 (70–80%), with less frequent associations observed with *BRD3* (bromodomain-containing protein 3) (15%), *NSD3* (nuclear receptor binding SET domain protein 3) (6%), *ZNF532*/*ZNF592* (Z4 zinc-finger protein family) (2%), and unidentified partners (7%) [14,15,16,17]. However, diffuse positivity on NUT IHC staining does not automatically confirm NUT carcinoma, as *CIC*::*NUTM1* sarcoma cases have also been reported since 2018 [18]. To further confirm the diagnosis, we performed FISH to analyze the *CIC*::*NUTM1*-rearrangement, which yielded negative results (Supplementary Figure S2). The differential diagnosis of *CIC*::*NUTM1* sarcoma relies on certain key features: (1) younger age of onset, (2) higher likelihood of central nervous system involvement, (3) absence of abrupt squamous differentiation, and (4) immunohistochemical expression of ETS variant transcription factor 4 (ETV4) as well as vimentin with negative p63 and p40 [19]. In our case, these criteria also support the diagnosis of NUT carcinoma.

Primary uterine NUT carcinoma can be misdiagnosed as other undifferentiated uterine tumors owing to its rarity and histological overlap. Considering the diagnostic challenge, NUT IHC staining and molecular testing for *NUTM1* rearrangement should be considered in undifferentiated uterine tumors with ambiguous histopathological features.

## Figures and Tables

**Table 1:** Immunohistochemical details.

Proteins	Results	Significance
NUT	Positive (diffuse)	Strong diffuse positivity is diagnostic for NUT carcinoma.
AE1/AE3	Negative	Positivity indicates the epithelial origin of the tumor.
p40	Negative	Specific marker for squamous cell carcinoma.
p63	Positive (rare)	Marker of squamous and myoepithelial differentiation.
β-catenin	Negative	Nuclear positivity is observed in Wnt pathway-activated tumors, such as desmoid-type fibromatosis or solid-pseudopapillary neoplasm.
cyclin D1	Positive	Overexpression is associated with cell cycle progression, as observed in various malignancies, including sarcomas.
ER	Positive	Estrogen receptor positivity suggests hormone responsiveness, as usually observed in gynecologic tumors.
PgR	Positive	Progesterone receptor positivity; usually co-expressed with ER and supports hormone sensitivity.
WT-1	Positive (focal)	WT-1 is expressed in ovarian serous tumors and some mesothelial and stromal tumors.
desmin	Positive (rare)	Marker of muscle differentiation; rare positivity may indicate limited myogenic features.
αSMA	Negative	Expressed in smooth muscle and myofibroblast differentiation.
CD10	Negative	Usually positive in endometrial stromal sarcoma and some renal tumors.
CD34	Negative	Typically positive in vascular tumors.
myogenin	Negative	Myogenic regulatory factor; negativity rules out rhabdomyosarcoma.
INSM1	Negative	Sensitive marker for neuroendocrine differentiation.
S100	Negative	Marker of neural or melanocytic origin.
CD99	Positive (focal)	Expressed in Ewing sarcoma and other small round-cell tumors.
NKX2	Negative	Expressed in Ewing sarcoma.
SALL4	Negative	Marker for germ cell tumors.
BCOR	Non-specific	Nuclear expression may indicate *BCOR*-rearranged sarcomas; interpretation should be carefully performed.
inhibinα	Negative	Marker of sex cord-stromal differentiation; negativity argues against such origin.
Melan A	Negative	Melanocytic marker; negative in non-melanocytic tumors.
HMB45	Negative	Marker of melanocytic differentiation.
CD45	Negative	Leukocyte common antigen; negativity rules out hematolymphoid origin.
CD117	Negative	Positive in GIST and some germ cell tumors.
ARID1A	Retained	Retained expression suggests no loss-of-function mutation in the SWI/SNF chromatin remodeling complex.
ARID1B	Retained	
INI-1	Retained	
BRG1	Retained	
PMS2	Retained	Mismatch repair proteins; retained expressions suggest microsatellite stability.
MSH6	Retained	
Ki-67	70%	Proliferation index: higher values suggest aggressive behavior.

**Table 2:** Six cases of NUT carcinoma in pelvic gynecological organs.

Case	Author	Year	Age, Sex	Clinical Primary Site	Pelvic Gynecological Lesion	Gene Fusion	IHC	Initial Serum CA125	Surgery	Chemotherapy/ Radiotherapy	Progression-Free Survival	Overall Survival
1	Jiang et al. [7]	2023	53, F	Ovary	Bilateral ovary	*MXI1*::*NUTM1*	NUT(+), ER/PgR(+), p40(−), p63(−), Pan CK(−)	469	Diagnostic laparoscopy; IDS (TAH, bilateral SO, omentectomy, PLN, small intestine resection)	3 cycles of Paclitaxel, Carboplatin, and Bevacizumab (NAC) <SD>	6 months	8 months (DOD)
2	Jung et al. [4]	2021	54, F	Ovary	Bilateral ovary involving uterus (12 × 12 cm)	–	NUT(+), ER/PgR(+), p40(−), p63(−), Pan CK (faint +), Vimentin (focal +)	502	–	2 cycles of Bleomycin, Etoposide, and Cisplatin <PD>	2 months	NA
3	Stevens et al. [8]	2019	32, F	Ovary and lung	Ovary	*BRD4*::*NUTM4*	NUT(+), AE1/AE3(+)	NA	NA	NA	–	NA
4	Ball et al. [9]	2012	19, F	Ovary and lung	Left ovary (15 × 12 cm)	*BRD4*::*NUTM4*	NUT(+), p63(+), WT-1(−)	NA	–	4 cycles of Bleomycin, Etoposide, and Cisplatin <PD>	–	5 months (DOD)
5	Higashi-no et al. [10]	2022	22, F	Supraglottis	Right ovary (12 cm)	–	NUT(+), p40(+), AE1/AE3(+), CK20(−)	125	Right SO	Chemoradiotherapy (Cisplatin) <PD>; Cetuximab and Paclitaxel <PD>; Nivolumab <PD>	0.5 months	7.5 months (DOD)
6	Dragoescu et al. [11]	2015	38, F	Lung	Bilateral ovary (left 10 cm, right 2.7 cm)	–	NUT(+), ER(−), p63(+), CK5/6(+), CK7 (focal +), CK20(−)	88	Video-assisted right pleural biopsy; bilateral SO	Whole-brain external beam radiotherapy	–	2.5 months (DOD)

## Data Availability

The data presented in this study are available on request from the corresponding author due to privacy.

## Supplementary Materials

The following supporting information can be downloaded at: https://www.mdpi.com/article/10.3390/clinpract16010020/s1, Figure S1: PRISMA flow chart for literature search; Figure S2: Negative results for the *CIC*-break-apart on fluorescence in situ hybridization (FISH). All the red/green signals are either overlapping or highly adjacent, indicative of an absence of *CIC*-rearrangement. We used the SPEC CIC Dual Color Break Apart Probe (ZytoVision GmbH, Bremerhaven, Germany) according to the manufacturer’s instructions, which was labeled in red on the 5′-side and green on the 3′-side of *CIC* (19q13.2).

## Abbreviations

The following abbreviations are used in this manuscript:

NUT	Nuclear protein in testis
*NUTM1*	*NUT midline carcinoma family member 1*
IHC	Immunohistochemical
MRI	magnetic resonance imaging
DWI	Diffusion-weighted imaging
FISH	Fluorescence in situ hybridization
FDG-PET	Fluorodeoxyglucose positron emission tomography
