# Supplementary figures and images for: Primary Uterine NUT Carcinoma: A Case Report and Literature Review
Source: Clin Pract. 2026 Jan 21;16(1):20. doi: 10.3390/clinpract16010020 (PMC12839556; doi:10.3390/clinpract16010020)

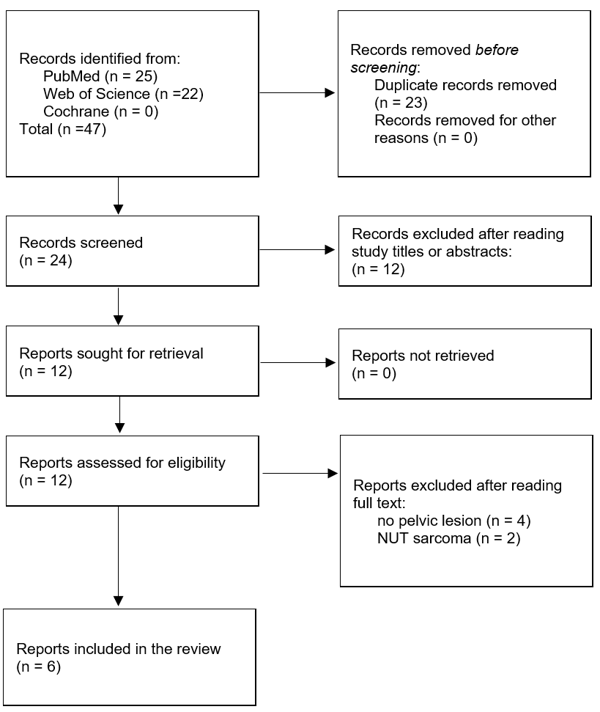

Supplement: Supplementary file 1 [file clinpract-16-00020-s001.zip › Supplementary_Figure1.tif]

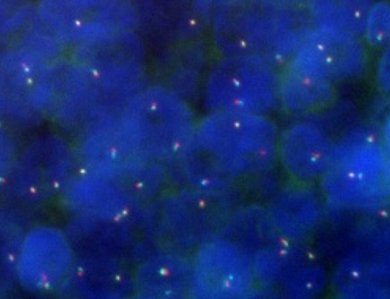

Supplement: Supplementary file 1 [file clinpract-16-00020-s001.zip › Supplementary_Figure2.tif]
